# Comprehensive identification and expression analysis of CRY gene family in *Gossypium*

**DOI:** 10.1186/s12864-022-08440-9

**Published:** 2022-03-24

**Authors:** Chaochen Huang, Pengbo Li, Junfeng Cao, Zishou Zheng, Jinquan Huang, Xiufang Zhang, Xiaoxia Shangguan, Lingjian Wang, Zhiwen Chen

**Affiliations:** 1 National Key Laboratory of Plant Molecular Genetics and National Center for Plant Gene Research, Institute of Plant Physiology and Ecology/CAS Center for Excellence in Molecular Plant Sciences, Chinese Academy of Sciences, Shanghai, 200032 China; 2 School of Life Science and Technology, ShanghaiTech University, Shanghai, 201210 China; 3 University of Chinese Academy of Sciences, Shanghai, 200032 China; 4 Shanxi Key Laboratory of Cotton Germplasm Resources Utilization and Molecular Design Breeding, Institute of Cotton Research, Shanxi Agricultural University, Yuncheng, 044099 China; 5 Institute of Carbon Materials Science, Shanxi Datong University, Datong, 037009 China

**Keywords:** Cryptochrome, Cotton, Phylogenetics, Organ-specific expression, Abiotic stress

## Abstract

**Background:**

The cryptochromes (CRY) are specific blue light receptors of plants and animals, which play crucial roles in physiological processes of plant growth, development, and stress tolerance.

**Results:**

In the present work, a systematic analysis of the CRY gene family was performed on twelve cotton species, resulting in 18, 17, 17, 17, and 17 CRYs identified in five alloteraploid cottons (*Gossypium hirsutum*, *G. barbadense*, *G. tomentosum*, *G. mustelinum* and *G. darwinii*), respectively, and five to nine CRY genes in the seven diploid species. Phylogenetic analysis of protein-coding sequences revealed that *CRY* genes from cottons and *Arabidopsis thaliana* could be classified into seven clades. Synteny analysis suggested that the homoeolog of *G. hirsutum Gh_A02G0384* has undergone an evolutionary loss event in the other four allotetraploid cotton species. *Cis*-element analysis predicated the possible functions of *CRY* genes in *G. hirsutum*. RNA-seq data revealed that *Gh_D09G2225*, *Gh_A09G2012* and *Gh_A11G1040* had high expressions in fiber cells of different developmental states. In addition, the expression levels of one (*Gh_A03G0120*), 15 and nine *GhCRY* genes were down-regulated following the PEG, NaCl and high-temperature treatments, respectively. For the low-temperature treatment, five *GhCRY* genes were induced, and five were repressed. These results indicated that most *GhCRY* genes negatively regulate the abiotic stress treatments.

**Conclusion:**

We report the structures, domains, divergence, synteny, and *cis*-elements analyses systematically of *G. hirsutum CRY* genes. Possible biological functions of *GhCRY* genes in differential tissues as well as in response to abiotic stress during the cotton plant life cycle were predicted.

**Supplementary Information:**

The online version contains supplementary material available at 10.1186/s12864-022-08440-9.

## Background

Cryptochromes (CRYs) are found in archaea, bacteria, algae, terrestrial plants, and humans, and they are photoreceptors for plants and animals [1–3]. Cryptochromes were first identified in *Arabidopsis thaliana*, named as HY4 or CRY1, which encodes a DNA photolyase protein responsible for the blue-light inhibition of hypocotyl elongation [4, 5]. Cryptochromes contain two-domain structure: the highly conserved flavin adenine dinucleotide (FAD)-binding photolyase homology region (PHR) domain and the divergent CRY C-terminal extension (CCE) domain [6, 7].

The numbers of cryptochromes vary among plant species, ranging from three in Arabidopsis to seven in soybean [8, 9]. All higher plants studied to date have two phylogenetically diverged clades of cryptochromes, CRY1 and CRY2 [10, 11]. Most cryptochromes in plants are involved in regulation of gene expressions of the plant life cycle [12–14]. Among them, CRY1 could inhibit the hypocotyl elongation in *Arabidopsis* [4], and the grain dormancy and germination in barley [15]. *Arabidopsis* CRY1 also controls photomorphogenesis through the regulation of H2A.Z deposition [16]. CRY2 could interact with CIB1 or SPA1 to regulate the floral initiation in *Arabidopsis* [17, 18], and suppress the leaf senescence in soybean [19]. Furthermore, CRY1 and CRY2 act together to stimulate the stomata opening and development in *Arabidopsis* [20, 21].

Among the many processes regulated by cryptochromes, responses to biotic and abiotic stresses, such as drought, salinity, heat, and so on, are one of the most active research topics in plant biology [22]. It has been demonstrated that cryptochromes in *Arabidopsis* enhance plant resistance to *Pseudomonas syringae* [23] and drought [21]. In tomato, CRY1a could modulate water deficit and osmotic stress responses [24] as well as mediate long-distance signaling of soil water deficit [25]. In rice, suppression of CRY1b improved salt tolerance as a result of down-regulation of the melatonin and brassinosteroid biosynthetic genes [26]. Overexpressing the wheat cryptochromes *TaCRY1a* and *TaCRY2* in *Arabidopsis* led to higher sensitivity to salt stress in the transgenic plants [27]. In addition, the *cry1* mutant of *A. thaliana* had a greater germination and seedling survival rate than the WT in salt-stressed conditions, and the mutant plants exhibited enhanced tolerance to salinity [28]. Obviously, these results support a role of cryptochromes acting as a negative regulator in plant response to salinity.

The cotton genus *Gossypium*) contains more than 50 species, of which the cultivated species are the most important fiber crops in the world [29–31]. Recent advances in cotton genomics have produced the resources necessary to analyze gene families in *Gossypium.* Multiple high-quality genome sequences are available for several species, including diploid species, i.e., *Gossypium thurberi* (D_1_), *G. raimondii* (D_5_), *G. turneri* (D_10_) [32–35], *G. herbaceum* (A_1_; cultivated), *G. arboreum* (A_2_; cultivated) [36–38], *G. longicalyx* [39], *G. australe* [40] and tetraploid *G. hirsutum* (AD_1_; cultivated), *G. barbadense* (AD_2_; cultivated), *G. tomentosum* (AD_3_), *G. mustelinum* (AD_4_) and *G. darwinii* (AD_5_) [38, 41–48], and *Gossypium* sister genera *Gossypioides kirkii* [49]. These genome sequences generate an excellent platform for dissecting gene functions by forward and reverse genetics, and for molecular breeding. Although genome sequencing has facilitated the functional characterizations of cotton genes, the *CRY* family genes in *Gossypium* have not been extensively explored.

In the current study, we performed a genome-wide screening of *CRY* genes in cottons, based on data gathered from recent whole-genome sequencing results. We used in silico approach to identify *CRY* genes in *Gossypium* species, focusing gene structures, conserved domains, synteny, *cis*-elements, and the phylogenetic relationships. Moreover, the tissue-specific expression patterns and the transcriptional responses of *GhCRYs* to abiotic stresses were examined. Our data provide inspirations for further research of cotton CRYs, as well as for molecular design of cotton cultivars with desired traits.

## Results

### Identification and chromosomal location of CRY family genes in *G. hirsutum*

**Fig. 1:**
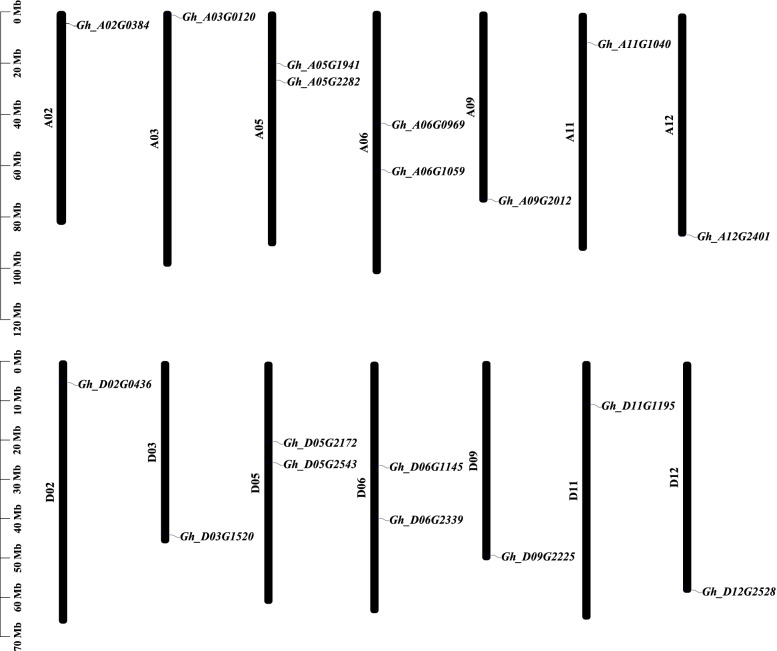
Dispersed distribution of *CRY* genes in *G. hirsutum* (AD_1_) chromosomes. 18 *GhCRY* genes are scattered over 14 of the 26 *G. hirsutum* chromosomes

Cryptochromes are a class of photolytic flavin proteins, which act as UV-A/blue light receptors and play an important role in plant growth and development [50]. These proteins are defined by the presence of a FAD-binding domain of DNA photolyase [51, 52]. Hmmersearch against the *G. hirsutum* genome database with the conserved domains (PF00875 for DNA photolyase domain) identified 18 *CRY* genes (Table 1), which are dispersed over 14 of the 26 *G. hirsutum* chromosomes, with most, but not all, homoeologs conserved in the two (A and D) subgenomes (Fig. 1).

**Table 1:** Sequence characteristics of *GhCRY* (*Gossypium hirsutum* cryptochrome) genes and proteins

Locus Name	Chr	Genomics Position	CDS	No. of Introns	Size (aa)	DNA Photolyase Domain	FAD binding 7 Domain	Cryptochrome C Domain	Hydrolase 4 Domain	MW	pI
Gh_A02G0384	A02	4,840,292–4,843,745	2,268	4	755	6–169	285–485			85.81	6.26
Gh_D02G0436	D02	5,763,605–5,767,015	2,268	4	755	6–170	285–485			85.49	6.41
Gh_A03G0120	A03	1,871,238–1,873,553	1,497	8	498	34–200				57.36	8.89
Gh_D03G1520	D03	44,312,391–44,314,703	1,497	8	498	34–200				57.38	8.89
Gh_A05G1941	A05	20,388,600–20,391,811	2,049	3	682	7–173	283–483	510–627		76.95	5.82
Gh_D05G2172	D05	20,346,471–20,349,578	2,049	3	682	7–173	283–483	510–627		76.98	5.55
Gh_A05G2282	A05	26,904,700–26,907,437	2,022	3	673	3–168	279–479	506–618		76.23	5.62
Gh_D05G2543	D05	25,786,819–25,789,574	2,022	3	673	3–168	279–479	506–618		76.17	5.62
Gh_A06G0969	A06	43,955,190–43,958,563	1,629	12	542	52–244	345–529			61.75	9.06
Gh_D06G1145	D06	26,445,874–26,448,973	1,380	10	459	82–268	368–456			52.29	9.43
Gh_A06G1059	A06	62,119,515–62,125,030	1,644	13	547	18–189	303–503			63.19	8.96
Gh_D06G2339	D06	67,821–73,312	1,644	13	547	18–188	303–503			63.32	8.97
Gh_A09G2012	A09	73,305,167–73,307,596	1,977	3	658	21–185	300–500			74.87	5.86
Gh_D09G2225	D09	49,444,930–49,447,313	1,935	3	644	7–171	286–486			73.59	6.08
Gh_A11G1040	A11	11,750,568–11,752,551	1,389	3	462	126–296				50.37	8.00
Gh_D11G1195	D11	11,089,391–11,091,357	1,392	3	463	126–296				50.63	7.52
Gh_A12G2401	A12	86,483,003–86,486,801	2,073	13	690	46–202			425–651	78.43	6.29
Gh_D12G2528	D12	58,181,188–58,184,994	2,073	13	690	46–195			425–650	78.35	7.63

*bp* Base pair, *Chr.* Chromosome, *aa* Amino acid, *MW* Molecular weight, *kDa* Kilodalton, *pI* Isoelectric point

### Structural organization of *GhCRY* genes

Less than two-fold variation in length was detected in the predicted coding sequences (CDS) for the recovered *GhCRY*s, from 1380 bp for *Gh_D06G1145* to 2,268 bp for *Gh_A02G0384/Gh_D02G0436* (Table 1), which translate to proteins ranging from 459 amino acids (aa) (52.29 kDa) to 755 aa (85.81 kDa). Predicted isoelectric points (pI) for members of this family vary widely, from 5.55 to 9.43. All of the putative GhCRY proteins have DNA photolyase domain in the N-terminal region (Table 1). Twelve GhCRY proteins have FAD binding 7 domain, four proteins have cryptochrome C domain and two have hydrolase 4 domain in the C-terminal region, respectively (Table 1).

**Fig. 2:**
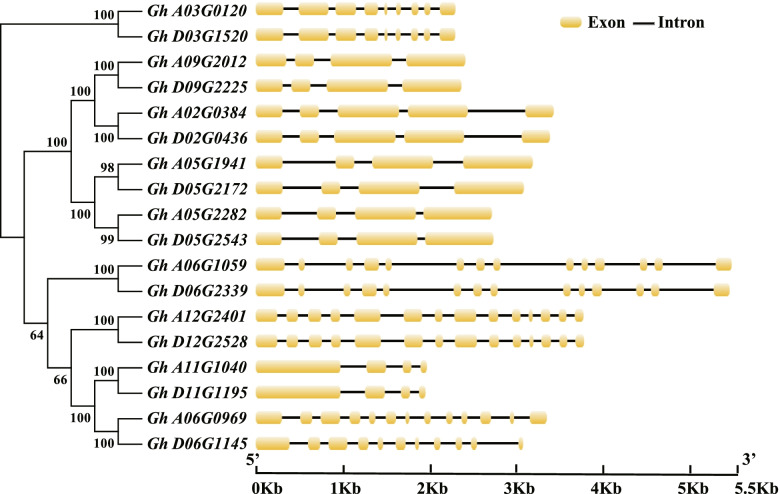
Phylogenetic tree and gene structure of *CRY* genes in *G. hirsutum*. Exons and introns are represented by yellow boxes and black lines, respectively

While all putative *GhCRY* genes contain introns (Fig. 2), they also exhibit considerable variations, in both length and number. In general, homoeologous *GhCRY* genes show highly similar intron patterns, however, among different homoeologous pairs the genes vary in both intron numbers (3 to 13) and lengths. One of the homoeologous gene pairs does exhibit divergence in structure, namely *Gh_A06G0969 vs Gh_D06G1145*, which contain 12 and 10 introns, respectively. Characterization of parental genes (both containing 12 introns in the diploids) for the homoeologs suggests that this structural variation was descendant divergence rather than inherited. In addition, phylogenetic relationship of the *GhCRY* gene family is not consistent with the intron/exon structures characterized (Fig. 2).

### Phylogenetic analysis of *CRY* family genes in *Gossypium*

**Fig. 3:**
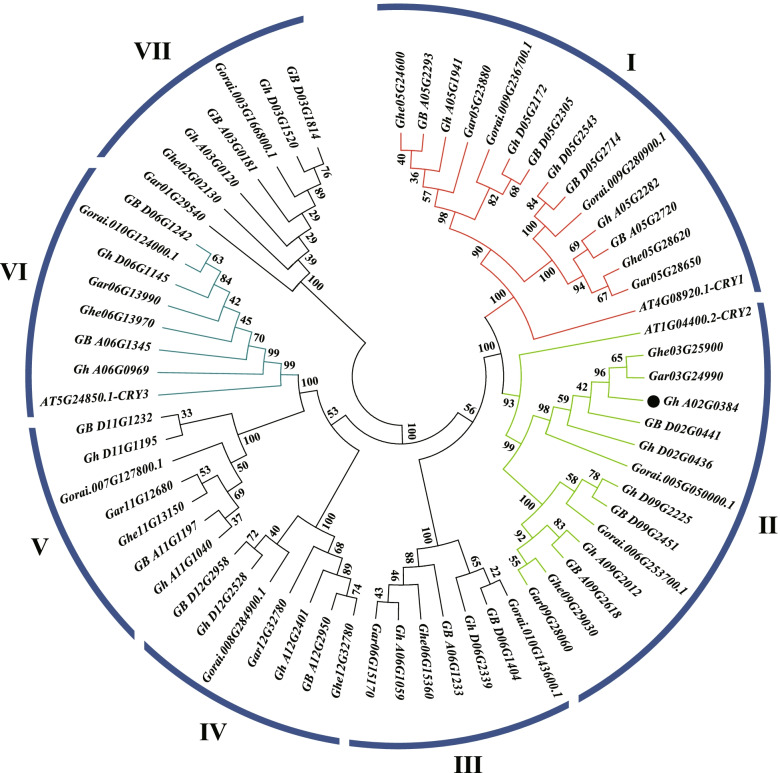
Phylogenetic analysis of *CRY* genes from five *Gossypium* species and *Arabidopsis thaliana*. The phylogenetic tree was established with entire protein-coding sequences with NJ methods. The numbers on the branches indicate bootstrap support values from 1000 replications

The general but incomplete conservation of *CRY* genes between the two subgenomes of *G, hirsutum* prompted us to ask whether the minimal loss and/or gain occurred before or after the marriage of the two diploid progenitors. We specifically assessed this using the protein-coding sequences of 62 cotton *CRY* genes (*G. hirsutum*, 18; *G. barbadense*, 17; *G. raimondii*, 9; *G. arboreum*, 9; and *G. herbaceum*, 9) with 3 *Arabidopsis thaliana CRY* genes for phylogenetic analysis (Fig. 3). Seven clades (I–VII) were robustly supported with each of the *A. thaliana* genes associated with clades I, II and VI, respectively, and the reamining four clades were composed of *Gossypium CRY* genes only.

Overall, the expected diploid-polyploid topology is reflected in the tree for each set of orthologous/homoeologous genes, indicating general preservation during diploid divergence and through polyploid evolution. That is, the number of *CRY* genes in tetraploids was generally additive with respect to the model diploid progenitors, with each homoeolog (A_t_ or D_t_) sister to their respective counterparts in the diploid species. Clades I and II had the most *CRY* genes, and the other five clades contained equal numbers (Fig. 3). In clades I and II, genes related to *AtCRY1* and *AtCRY2* exhibit duplications in *Gossypium* species, which indicate a duplication event in *Gossypium* compared to *A. thaliana*. In addition, the *Gossypium CRY* genes of clade I have a sister relationship with *AtCRY1*, and clade II have the closest relationship with *AtCRY2*, and clade VI was classified with *AtCRY3*. Therefore, it is speculated that the function of *CRY* genes in these clades of cotton is similar to their homologs in *Arabidopsis*.

Although the *CRY* family exhibits general conservation, a few deviations were noted. For example, Clade II exhibits evidence of homoeolog loss; that is, the A_t_ copy of *GB_D02G0441* is missing from *G. barbadense* genome, whereas both copies (*Gh_A02G0384*/*Gh_D02G0436*) exist in *G. hirsutum*. This gene loss might specific to *G. barbadense* after divergence of the two allotetraploid species.

### Divergence of *CRY* genes in allotetraploid *G. hirsutum* and its diploid progenitors

**Fig. 4:**
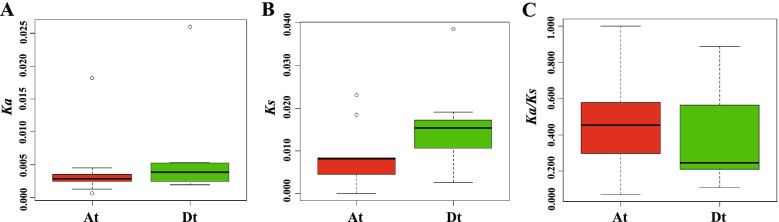
Distribution of Ka (**A**), Ks (**B**) and Ka/Ks (**C**) values of CRY genes between the A and D subgenomes versus their corresponding diploid progenitor homoeologs

The *CRY* genes in the two diploid species were then compared with *G. hirsutum* A_t_- and D_t_-subgenome homoeologs (Fig. 4, Additional file 1: Table S1). To explore the evolutionary relationship and possible functional divergence of *CRY* genes between the allotetraploid cotton and their extent putative diploid progenitors, the nonsynonymous substitution (*Ka*) and synonymous substitution values (*Ks*) and the *K*a/*K*s ratios for each pair of the genes were calculated (Additional file 1: Table S1). By comparing the *Ka* and *Ks* values of 18 orthologous gene sets between the allotetraploid and the respective diploid genomes, we found that the *Ka* and *Ks* values are higher in the D_t_ subgenome than in the A_t_ subgenome (Fig. 4a, b). These results indicate that *GhCRY* genes in the D_t_ subgenome tend to have experienced faster divergence than their A_t_ counterparts. However, the *Ka/Ks* ratios of D_t_ subgenome was lower than that of A_t_ subgenome (Fig. 4c), indicating that *GhCRY* genes in A_t_ subgenome were subjected to positive selection during the course of evolution and domestication, and might have resulted in diverged functions.

### Dynamic evolution of *CRY* family genes in *Gossypium*

**Fig. 5:**
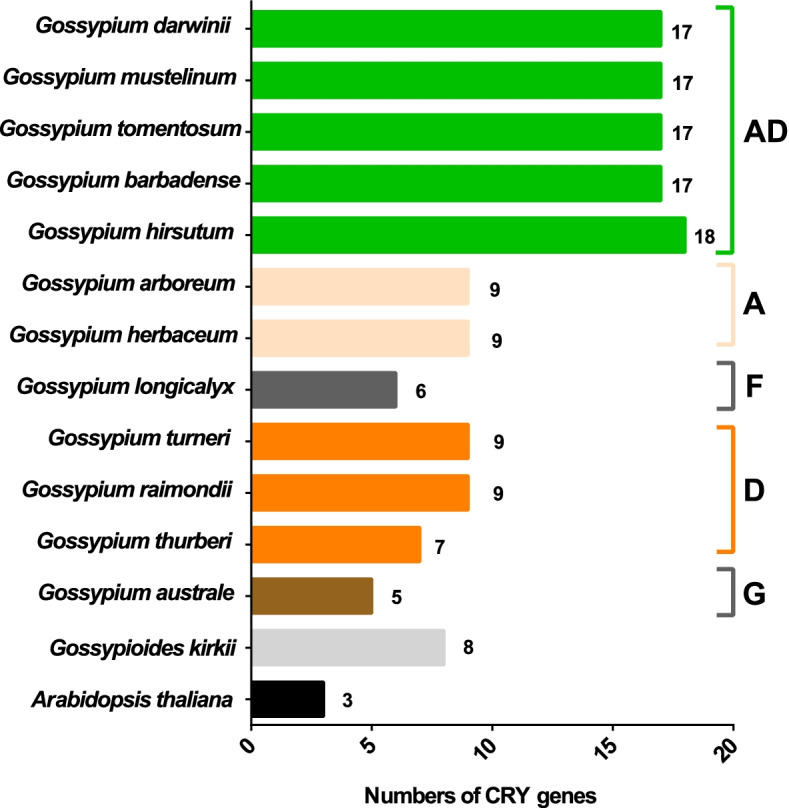
Dynamic evolution of the number of *CRY* family genes in 12 *Gossypium* species, *Gossypium kirkii* and *Arabidopsis thaliana*

We further evaluated the general preservation of *CRY* genes in 12 *Gossypium* species, *Gossypioides kirkii* and *Arabidopsis thaliana* (Fig. 5). In *A. thaliana* only three *CRY* genes were identified, whereas and the relative of *Gossypium*, *Gossypioides kirkii*, has eight. All *Gossypium* species surveyed recovered a minimum of five putative *CRY* genes in *G. australe* (G_2_) to 18 in *G. hirsutum* (AD_1_). Among the D genome species, *CRY* gene copy number varied from a minimum of 7 in *G. thurberi* (D_1_), to 9 in both *G. raimondii* (D_5_) and *G. turneri* (D_10_). The two cultivated A-genome species of *G. herbaceum* (A_1_) and *G. arboreum* (A_2_) also have 9 copies. The sister-species of A-genome, *G. longicalyx* (F_1_), contains 6 *CRY* genes. The *CRY* copy numbers in the allotetraploid species surveyed varied from 17 in four species: *G. barbadense* (AD_2_), *G. tomentosum* (AD_3_), *G. mustelinum* (AD_4_) and *G. darwinii* (AD_5_), to 18 in *G. hirsutum* (AD_1_). Notably, this high copy number in tetraploid is slightly more than double the copy number in diploid, likely reflective of the duplicated history of cotton. Comparatively, in *G. hirsutum* (AD_1_) the *CRY* copy number is stable after polyploidization, whereas the other four allotetraploid cottons included in this analysis all appear to have undergone a homoeolog loss (17 versus18), while *G. hirsutum* has retained it on the A02 chromosome (*Gh_A02G0384*) (as stated above, Fig. 3).

### Chromosomal distribution and synteny analysis of *Gossypium CRY* genes

**Fig. 6:**
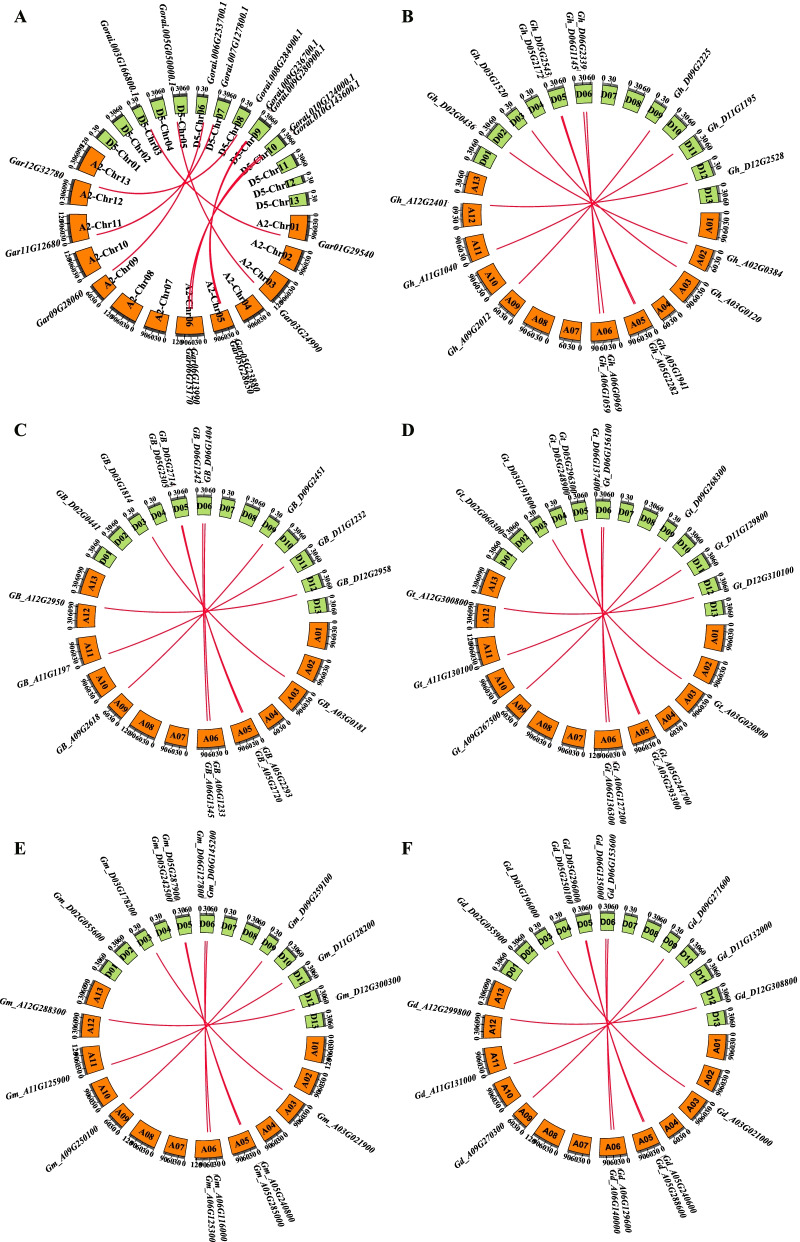
Syntenic analysis of the *Gossypium CRY* gene family. **A** *G. arboreum vs G. raimondii*; **B** *G. hirsutum*; **C** *G. barbadense*; **D** *G. tomentosum*; **E** *G. mustelinum*; **F** *G. darwinii*. The scale on the circle is in Megabases. The *CRY* gene IDs of each *Gossypium* species were on the chromosomes; the numbers of each chromosome of *Gossypium* species are shown inside the circle of each bar. The syntenic relationships of *CRY* gene are connected by red lines

Based on these *Gossypium* genomes, the location of *CRY* genes and the length of chromosomes from two diploid species and five allotetraploid species were used to analyze the chromosomal distribution and synteny (Fig. 6). High similarity was found in the chromosomal distribution patterns among these seven cotton species. The *CRY* genes were unevenly distributed on chromosomes with divergence detected between the diploid and allotetraploid species. For instance, no *CRYs* were found on Chr 02 of two A-genome species nor on Chr A02 of the allotetraploid specie of *G. barbadense* (AD_2_), *G. tomentosum* (AD_3_), *G. mustelinum* (AD_4_) and *G. darwinii* (AD_5_), except *G. hirsutum* (AD_1_) which has one (*Gh_A02G0384*) on this chromosome (Fig. 6A). There were totally 104 *CRYs* distributed throughout the 80 chromosomes, comprising 38 located on the A or A_t_ (sub)genomes and 42 located on the D or Dt (sub)genomes. The majority of *CRYs* were located on the proximate or the distal ends of the chromosomes. In addition, there were nine collinear gene pairs between *G. raimondii* and *G. arboreum*, nine between A_t_ and D_t_ subgenomes of *G. hirsutum*, and eight for the other four allotetraploid subgenomes.

### *Cis*-element analysis of *CRY* genes in *G. hirsutum*

*Cis*-elements are involved in the responsive to corresponding stimulations to regulate the expression of genes [53]. In this study, a 1.5-kb fragment upstream to the start codon of each *CRY* gene of *G. hirsutum* was extracted to investigate putative *cis*-elements in the mediation of gene expression using the PlantCARE server [54]. In total 581 *cis*-elements among 18 *GhCRY* genes were identified, ranging from 22 in *Gh_A02G0384* to 47 in *Gh_D03G1520* (Fig. S1). Some *cis*-elements were predicted to mediate the phytohormone (ABRE) and stress (TC-rich repeats) responses (Fig. S1). 13 *GhCRY* gene promoters possess at least one abscisic acid responsiveness element (ABRE), and 11 *GhCRY* genes have at least one antioxidant response element (ARE). There were 9 *GhCRYs* which harbor the elements involved in the MeJA-responsiveness (CGTCA-motif and TGACG-motif), four have the *cis*-acting element involved in salicylic acid responsiveness (TCA element), two have gibberellin-responsive element (GARE-motif), and three have auxin-responsive element (TGA-element and AuxRR-core). Of the 18 *G. hirsutum CRY* genes (*GhCRY*s), all have light responsiveness element (Box 4 and G box) and at least two MYB binding site elements (MYB), three have meristem expression element (CAT-box) and four (*Gh_D02G0436*, *Gh_D03G1520*, *Gh_A05G2282* and *Gh_D05G2543*) have low-temperature responsiveness element (LTR). The remaining elements related to stress, such as thoese of defense and stress responsiveness (TC-rich repeats), W-box recognized by WRKY transcription factors, wound-responsive element (WUN-motif), and MYB-binding site involved in drought inducibility (MBS), were also detected.

### Expression patterns of *GhCRY* genes in different *G. hirsutum* tissues

**Fig. 7:**
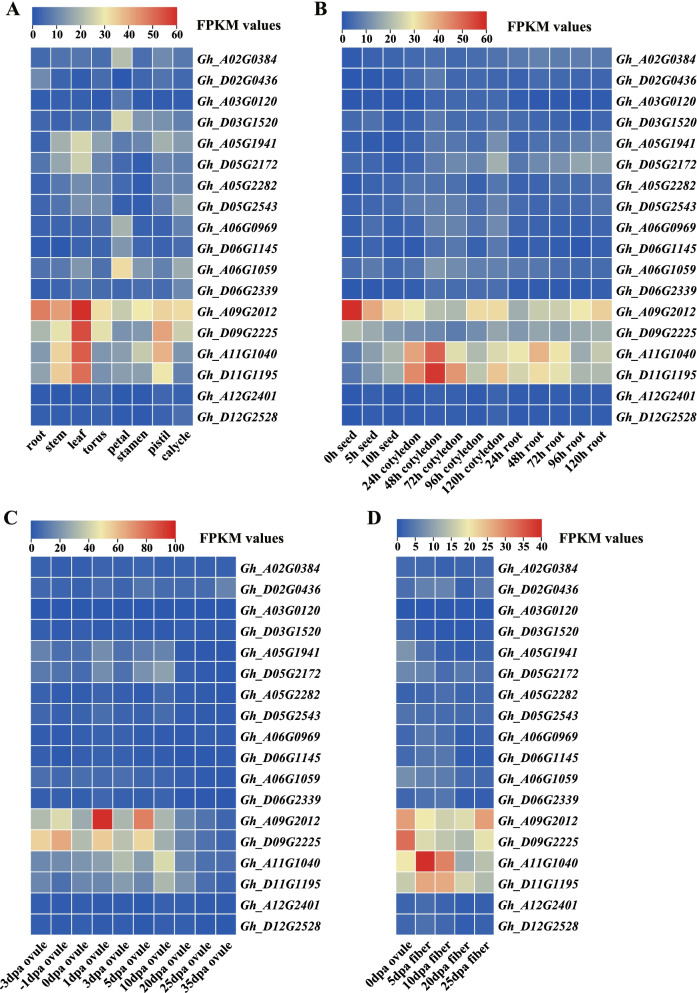
Expression patterns of *GhCRY* genes in different cotton tissues and fiber cells of different stages based on the RPKM values of RNA-seq data. **A** Expression profiles of *GhCRY* genes in eight cotton tissues. **B** Expression patterns of *GhCRY* genes in seed germination, cotyledons and roots after germination. **C** Expression patterns of *GhCRY* genes in ovules of different stages. **D** Expression patterns of *GhCRY* genes in fibers of different stages

The expression profile of a gene family can provide valuable clues to the possible functions of the gene. Analysis of 18 *GhCRY*s showed that most genes differ in spatial expression patterns. For instance, the expression levels of *Gh_A09G2012*, *Gh_D09G2225*, *Gh_A11G1040* and *Gh_D11G1195* in root, stem, leaf, torus, stamen, pistil and calycle were significantly higher than those of other *GhCRY* genes (Fig. 7a). *Gh_A06G1059* presented the highest expression level in petal (Fig. 7a). In addition, four genes (*Gh_A09G2012*, *Gh_D09G2225*, *Gh_A11G1040* and *Gh_D11G1195*) also showed high expression in seed, root and cotyledon samples at different time points post seed germination (Fig. 7b). In ovule samples of different developmental stages, *Gh_A09G2012* and *Gh_D09G2225* had the highest expression levels followed by *Gh_A11G1040* and *Gh_D11G1195* (Fig. 7c). In fiber samples of different developmental stages, *Gh_A09G2012* had the highest expression at 20- and 25-dpa (Fig. 7d), suggesting that this CRY gene might play a role at the cell wall thickening stage. *Gh_D09G2225* was preferentially expressed in 0-dpa ovule. These two GhCRYs are homologous to *Arabidopsis AtCRY2* (Fig. 3). *Gh_A11G1040* showed the highest expression in 5- and 10-dpa fiber cells, which suggests that it may play a role at the elongation stage of fiber development (Fig. 7d). These three genes could be taken as candidate genes for subsequent transformation experiments to dissect their functions in cotton fiber development.

### Expression changes of *GhCRY* genes in* G. hirsutum* under different stresses

**Fig. 8:**
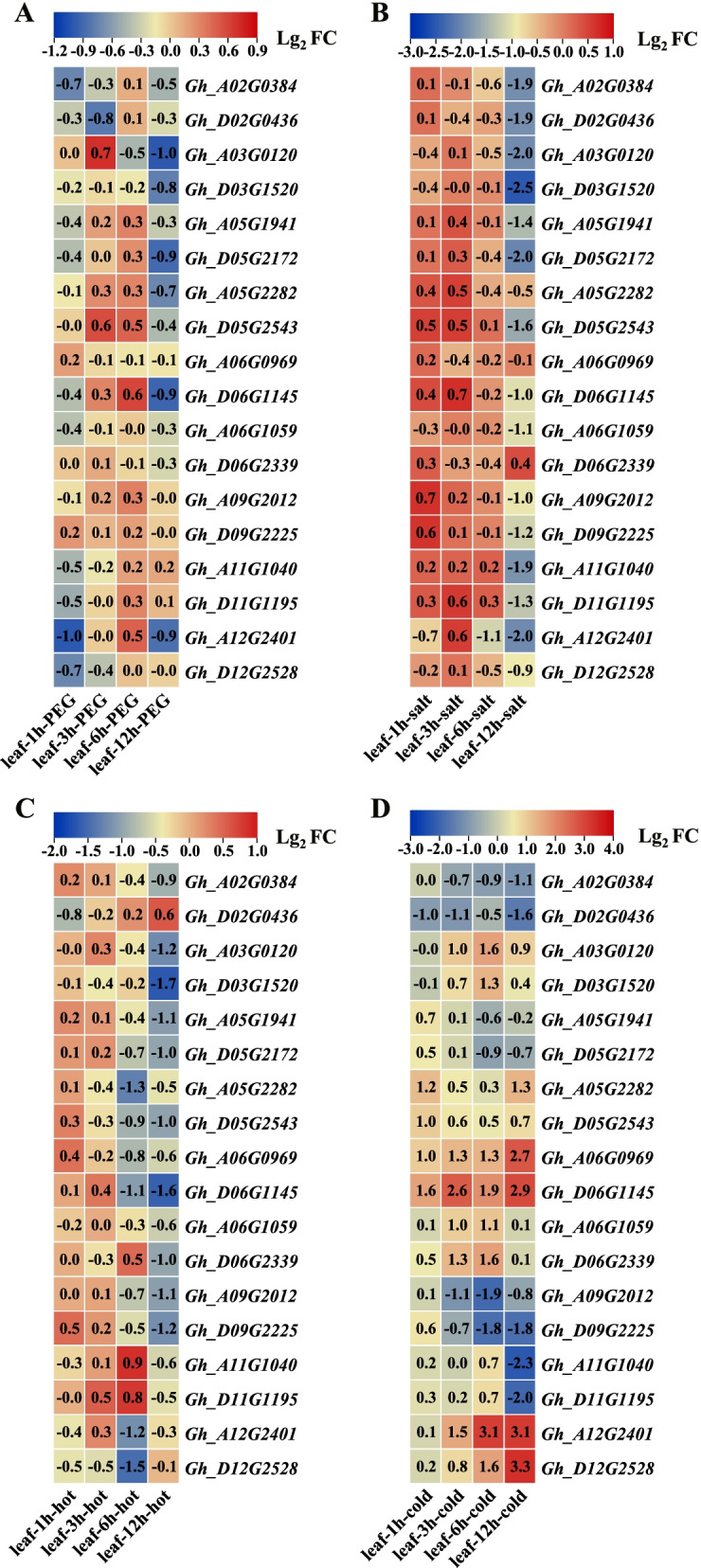
Expression patterns of *GhCRY* genes in response to different stresses from RNA-seq data. The RNA-seq data were downloaded from Zhang et al., 2015 and re-analyzed the RPKM values of five time points (0, 1, 3, 6 and 12 h) after stresses treatments. **A** Drought stress; **B** Salt stress; **C** Hot stress; **D** Cold stress

Cotton is often subjected to a variety of abiotic stresses during its growth and development. Therefore, we analyzed the expression changes of *CRY* genes under simulated drought (PEG 6000), salt (NaCl), heat and cold abiotic stresses from RNA-seq data (Fig. 8). At different time points of PEG6000 simulation drought condition, expressions of most *GhCRY* genes were not changed (|lg_2_ (Fold change)|≥ 1| as the threshold of differentially expressed genes). For instance, the expression of *GhCRY* genes did not change after PEG treatment for 3 and 6 h (Fig. 8a). However, *Gh_A12G2401* was down-regulated after 1 h of PEG treatment (Fig. 8a). After 12 h of PEG treatment, the expression of one *GhCRY* gene (*Gh_A03G0120*) was repressed (Fig. 8a). These results indicated that only a limited nimber of *GhCRY* genes responded to drought stress.

Under salt stress, the expressions of *GhCRY* gene did not change after 1, 3 and 6 h of NaCl treatment (Fig. 8b). However, a prolonged (12 h) NaCl treatment down-regulated 15 *GhCRY* genes (Fig. 8b), indicating that they were negatively related to salt stress.

At the four time points of high temperature stress, the expression of *GhCRY gene* did not change after 1 and 3 h of high temperature treatment (Fig. 8c). After 6 h under high temperature, the expression levels of four *GhCRY* genes (*Gh_A05G2282*, *Gh_D06G1145*, *Gh_A12G2401* and *Gh_D12G2528*) decreased (Fig. 8c), and after 12 h of high temperature the down-regulaged *GhCRY* genes increased to nine (Fig. 8c). Notably, *Gh_D06G1145* gene was inhibited at both 6 and 12 h, suggesting that this gene might be a key factor in adjusting the response to high temperature stress in *G. hirsutum*.

As for the four time points of low temperature stress, there were four *GhCRY* genes which were up-regulated after 1 h of low temperature treatment (Fig. 8d). And 3 h in low temperature the expressions of five *GhCRY* genes were induced, and two were reduced (Fig. 8d). After 6 h in low temperature the expression levels of eight *GhCRY* genes were elevated and two were declined (Fig. 8d). Finally, extending the low temperature treatment to 12 h resulted in five *GhCRY*s were up-regulated and five down-regulated (Fig. 8d). Among them *Gh_A06G0969* and *Gh_D06G1145*, both homologous to *AtCRY3*, were induced at all the four-time points under the low temperature condition, suggesting their involvement in plant toleranbce to low temperature stress.

To confirm the accuracy of these *GhCRY* candidates in response to these stresses, the expressions of four *GhCRY* genes (*Gh_A05G1941*, *Gh_A05G2282*, *Gh_A06G1059* and *Gh_A12G2401*) under hot, cold, salt and PEG stresses were examined by qPCR (Additional file 3: Figure S2). These four genes were mostly repressed under different stress conditions, which verified their expression patterns under stress detected by RNA-seq and qPCR data were generally congruent.

## Discussion

In plants the function of CRYs vary not only among individual CRY members but also among species. *Arabidopsis thaliana* has three CRYs, CRY1 and CRY2 are located in the nucleus and play a multifaceted role in various aspects of plant growth and development [1, 55]. For instance, CRY1 primarily regulates photomorphogenic responses related to the inhibition of hypocotyl elongation, anthocyanin accumulation and cotyledon expansion, while CRY2 plays a role in the hypocotyl inhibition, circadian clock and photoperiod-dependent flowering [56]. However, CRY3 is a DASH protein located in chloroplasts and mitochondria [51], which works to repair UV-damaged DNA in a light-dependent manner [57]. Overall, the cryptochrome-mediated photoresponses remain unclear with the existing differences in plant species as well as their physiological responses [58, 59].

It has been reported that plant cryptochromes were involved in the adversity stress response [22, 24, 60, 61]. In our study, all the *GhCRY* genes identified were negatively related to the PEG, NaCl and high temperature treatments. The negative regulation pattern of CRY factors in response to the drought, salt and osmotic stresses is paobably common in plant species. For instance, in *Arabidopsis* CRYs play an important role in drought stress tolerance [21]. Overexpressing the CRY1 protein in *Arabidopsis* resulted in excessive water loss whereas *cry1cry2* double mutant plants were clearly more drought-tolerant than the wild type. In addition, introducing *Triticum aestivum* CRYs (*TaCRY1a* and *TaCRY2*) into *Arabidopsis* plants reduced osmotic stress tolerance, including drought and salt stresses [27]. Meanwhile, overexpression of *Sorghum bicolor SbCRY1a* in *Arabidopsis* rendered the transgenic plants oversensitive to salt stress [28]. In tomato (*Solanum lycopersicum* L.), CRY1a modulated the water deficit response under osmotic stress conditions, further increasing tomato growth by reducing malondialdehyde (MDA) and proline accumulation [24]. In addition, the tomato *cry1a* mutant plants showed enhance drought tolerance wing to the increased leaf relative water content [25]. In *Brassica napus* overexpressing *CRY1* resulted in plants that were very sensitive to osmotic stress, whereas the antisense silencing plants were more tolerant [60]. However, the relationship between CRYs and abscisic acid (ABA), and the role of other blue light photoreceptors in modulating water loss under drought or salt stresses is still unclear, thus further research is rewarding.

In the current study, nine *GhCRY* genes were found negatively related to the high temperature treatments, at least based on expression patterns. However, there were five *GhCRY* genes which were induced after a 12-h low temperature treatment, along with the repression of an equal number of the *GhCRY*s. These results demonstrated the functional divergence among the *CRYs* in *G. hirsutum*. Plant cryptochromes have been implicated in adaptations to the changing environmental factors, for instance, a report showed that low temperatures would increase the biological activity of CRY [62]. Consistently, as reported herein in cotton each CRYs may behave differently in response to low temperature stresses. Although there have been many studies on plant CRYs in regulating plant growth and development, as well as the response abiotic stresses, our understanding of cotton CRYs is still preliminary. This genome-wide survey paves the way for in-depth research of the function of cotton cryptochromes, which in turn will add valuable data to cotton breeding.

## Conclusions

We systematically analyzed cotton CRY family genes and their expressions using bioinformatic approaches. We analyzed gene structures, chromosomal locations, intron–exon organizations, phylogenetic relationships and expression patterns in different cotton tissues and under different stress conditions to predict their possible biological functions. In particular, the *GhCRY* highly expressed in cotton fiber cells were identified. The decreased expressions of several *GhCRY* genes in response to multiple abiotic stress implies their involvement in the regulation of growth and development under the abiotic stress conditions. Together, our results provide candidate genes to facilitate the functional identification of the *CRY* genes in cotton that are important in modulating plant growth, development and stress tolerance.

## Methods

### Identification of CRY family genes and CRY proteins in diploid and tetraploid *Gossypium* species

We downloaded the genome sequences of cotton species from the CottonGen database [63], including *G. raimondii* [32], *G. herbaceum* [38], *G. arboreum* [36], *G. hirsutum* [41], *G. barbadense*, *G. tomentosum*, *G. mustelinum* and *G. darwinii* [38, 41–48]. To identify all putative CRY transcription factor proteins in each genome assembly, the CRY protein conserved domains (PF00875 for DNA photolyase) were used to develop a Hidden Markov Model [64] profile matrix via the hmmbuild program from the HMMER package [65] using default parameters. This HMM profile matrix was used in conjunction with hmmersearch with default parameters against these *Gossypium* genome databases to identify putative CRY genes (*GhCRY*s). Previously identified *CRY* gene sequences from *Arabidopsis thaliana* (*AtCRY*s) were retrieved from the TAIR database [66] for phylogenetic comparison. The presence of conserved domains in each *Arabidopsis* and *Gossypium* gene was verified using the SMART conserved domain search tool [67] and Pfam databases [68].

### Chromosomal location and gene structure analyses

Chromosomal locations for each of the above identified *GhCRYs* were extracted from the genome annotation gff3 file [41]. Chromosomal locations of the predicted *GhCRYs* were visualized using TBtools [69], and the exon–intron structure of each gene was displayed using the online tool GSDS 2.0 [70]. The number of amino acids, molecular weight (MW), and theoretical isoelectric point (pI) of putative *GhCRYs* proteins were determined using the ProtParam tool [71].

### Sequence alignment, Ka, Ks and phylogenetic analyses

Complete protein-coding sequences for *CRY* genes from *Gossypium* and *AtCRY* were aligned using MAFFT with the G-INS-i algorithm [72]. The nonsynonymous substitutions rate (Ka) and synonymous substitution rate (Ks) were calculated using the DnaSP 6.0 [73]. The NJ phylogenetic tree was constructed using MEGA version 6.0 [74] by sampling 1000 bootstrap replicates.

### Analysis of *Cis*-acting element in promoter regions of *GhCRYs*

The upstream sequences (1.5 kb) [75] of the *GhCRYs* genes were retrieved from *G. hirsutum* genome sequence based on the gene locations [41]. Then, the retrieved promoter sequences were submitted to PlantCARE [54] to identify the potential *Cis*-acting element.

### Chromosomal mapping and synteny analysis of *CRY* genes in diploid and allotetraploid *Gossypium* species

*CRY* genes were mapped on chromosomes using TBtools [69] software. Blastn was used to determine *CRY* gene synteny. Then, TBtools [69] software was applied to express the syntenic relationship of the homologous gene pairs.

### Expression patterns of *GhCRYs* in different tissues and stress conditions

Raw RNA-Seq data for *G. hirsutum* seed, root, stem, leaf, torus, petal, stamen, ovary, calyx, ovule (-3 dpa, -1 dpa, 0 dpa, 1 dpa, 3 dpa, 5 dpa, 10 dpa, 20 dpa, 25 dpa, 35 dpa) and fiber (5 dpa, 10 dpa, 20 dpa, 25 dpa) were downloaded from the NCBI Sequence Read Archive (PRJNA 248,163) [41], represented by one library each. Reads were mapped to the *G. hirsutum* genome [41] via HISAT2 software with default parameters, and read abundance calculated via StringTie [76, 77]. Read counts were normalized in R3.2 using RUVSeq [78] and the internal control reference gene *GhUBQ7*, which is detected at relatively constant levels across different cotton samples [79]. Potential batch-effects were corrected by an improved version of ComBat, ComBat-seq [80]. Gene expression was estimated by Ballgown [81], using fragments per kilobase million (FPKM) values to calculate the gene expression levels across libraries. Expression levels of *G. hirsutum* leaf RNA-Seq data (in FPKM) for each *GhCRY* gene under drought, salt, heat and cold stress (time points: 0, 1, 3, 6, 12 h) were retrieved from the ccNET database [82]. Genes were considered differentially expressed if expression varied more than two-fold change with a p-value of less than 0.05. TBtools [69] was used to display the gene expression patterns from the calculated FPKM values.

### Plant cultivation and stresses treatment

*G. hirsutum* cv. R15 [83], were planted in a controlled environment at 28 °C day/20 °C night, with a 16-h light/8-h dark photoperiod [84]. For stress treatments, 28-day old plants were treated with 200 mM NaCl, PEG6000, 42 °C (hot) and 4 °C (cold), respectively. Leaves from stress-treated plants were collected at 12 h post-stresses treatments for further expression analyses. All plant tissues were frozen in liquid nitrogen immediately after collection and stored at -80 °C until RNA extraction. These treatments were sampled with three biological repeats.

### RNA extraction, cDNA synthesis and qRT-PCR expression analyses

Total RNAs from cotton tissues were extracted using the RNAprep pure plant kit (TIANGEN, Shanghai, China)as described [85]. The resulting RNAs were treated with DNase I prior to synthesizing cDNA with oligo (dT) primers and M-MLV Reverse Transcriptase (Invitrogen); these products were diluted fivefold before use. For quantitative real-time PCR (qRT-PCR), Primer5 software was used to design gene-specific forward and reverse primers (Additional file 4: Table S2). Analyses were performed with SYBR-Green PCR Mastermix (TaKaRa) on a cycler (Mastercycler RealPlex; Eppendorf Ltd, Shanghai, China). The *G. hirsutum histone-3* (*GhHIS3*) genes were used as internal references [81, 86], and the relative amount of amplified product was calculated following the 2^−∆∆Ct^ method [87]. For the *G. hirsutum* samples, relative expression levels among different stresses were normalized by calibrating with the leaves sample from the wild plants. The leaves sample was washed with DEPC sterile water three times before extracting the RNA.

## Supplementary Information

**Additional file 1: Table S1.** Ka, Ks and Ka/Ks analyses of *GhCRY* from the A and D subgenomes compared with their corresponding progenitor homoeologs.

**Additional file 2: Figure S1.** Cis-elements in promoter regions of *GhCRY* genes.

**Additional file 3: Figure S2.** Expression profile analyses of four *GhCRY* genes under different stress treatments at the time points of 12h analyzed by qRT-PCR.(**A**): *Gh_A05G1941*;(**B**):*Gh_A05G2282*; (**C**):*Gh_A06G1059*; (**D**): *Gh_A12G2401*.

**Additional file 4: Table S2.** List of forward and reverse primers used for qRT-PCR analyses.

## Data Availability

The genome sequences of cotton species and the genome annotation gff3 file in this manuscript were downloaded from the CottonGen database (https://www.cottongen.org/data/download) [61]. Raw RNA-Seq data for *G. hirsutum* seed, root, stem, leaf, torus, petal, stamen, ovary, calyx, ovule and fiber were downloaded from the NCBI Sequence Read Archive (https://www.ncbi.nlm.nih.gov/bioproject/PRJNA248163) (NCBI Sequence Read Archive SRR1695173, SRR1695174, SRR1695175, SRR1695177, SRR1695178, SRR1695179, SRR1695181, SRR1695182, SRR1695183, SRR1695184, SRR1695185, SRR1695191, SRR1695192, SRR1695193,SRR1695194, SRR1768504, SRR1768505, SRR1768506, SRR1768507, SRR1768508, SRR1768509, SRR1768510, SRR1768511, SRR1768512, SRR1768513, SRR1768514, SRR1768515, SRR1768516, SRR1768517, SRR1768518 and SRR1768519) [30]. The conserved domain of CRY proteins (Pfam ID: PF00875) was downloaded from the Pfam databases (http://pfam.xfam.org/family/PF06507#tabview=tab3). All other data generated or analyzed during this study are included in this published article and its Additional files.

## Abbreviations

CRYs: Cryptochromes
DPA: Days post anthesis
FPKM: Fragments per kilobase of transcript per million mapped fragments
*G. herbaceum*: *Gossypium herbaceum*
*G. arboreum*: *Gossypium arboreum*
*G. longicalyx*: *Gossypium longicalyx*
*G. thurberi*: *Gossypium thurberi*
*G. raimondii*: *Gossypium raimondii*
*G. turneri*: *Gossypium turneri*
*G. australe*: *Gossypium australe*
*G. hirsutum*: *Gossypium hirsutum*
*G. barbadense*: *Gossypium barbadense*
*G. tomentosum*: *Gossypium tomentosum*
*G. mustelinum*: *Gossypium mustelinum*
*G. darwinii*: *Gossypium darwinii*
MW: Molecular weight
pI: Isoelectric point

## References

[1] Cashmore AR, Jarillo JA, Wu YJ, Liu D (1999). Cryptochromes: blue light receptors for plants and animals. Science.

[2] Han X, Chang X, Zhang Z, Chen H, He H, Zhong B, Deng XW (2019). Origin and evolution of core components responsible for monitoring light environment changes during plant terrestrialization. Mol Plant.

[3] Wang Q, Lin C (2020). Mechanisms of cryptochrome-mediated photoresponses in plants. Annu Rev Plant Biol.

[4] Ahmad M, Cashmore AR (1993). HY4 gene of A. thaliana encodes a protein with characteristics of a blue-light photoreceptor. Nature.

[5] Lin C, Ahmad M, Gordon D, Cashmore AR (1995). Expression of an Arabidopsis cryptochrome gene in transgenic tobacco results in hypersensitivity to blue, UV-A, and green light. Proc Natl Acad Sci U S A.

[6] Lin C, Robertson DE, Ahmad M, Raibekas AA, Jorns MS, Dutton PL, Cashmore AR (1995). Association of flavin adenine dinucleotide with the Arabidopsis blue light receptor CRY1. Science.

[7] Partch CL, Clarkson MW, Ozgur S, Lee AL, Sancar A (2005). Role of structural plasticity in signal transduction by the cryptochrome blue-light photoreceptor. Biochemistry.

[8] Zhang Q, Li H, Li R, Hu R, Fan C, Chen F, Wang Z, Liu X, Fu Y, Lin C (2008). Association of the circadian rhythmic expression of GmCRY1a with a latitudinal cline in photoperiodic flowering of soybean. Proc Natl Acad Sci U S A.

[9] Huang Y, Baxter R, Smith BS, Partch CL, Colbert CL, Deisenhofer J (2006). Crystal structure of cryptochrome 3 from Arabidopsis thaliana and its implications for photolyase activity. Proc Natl Acad Sci U S A.

[10] Lin C, Shalitin D (2003). Cryptochrome structure and signal transduction. Annu Rev Plant Biol.

[11] Cao S, He S, Lv H, Zhang J, Aslam M, Cheng H, Hu A, Cao G, Zhang X, Yu Y (2020). Genome-wide analysis of the cryptochrome gene family in plants. Trop Plant Biol.

[12] Wang Q, Zuo Z, Wang X, Liu Q, Gu L, Oka Y, Lin C (2018). Beyond the photocycle-how cryptochromes regulate photoresponses in plants?. Curr Opin Plant Biol.

[13] Ma D, Li X, Guo Y, Chu J, Fang S, Yan C, Noel JP, Liu H (2016). Cryptochrome 1 interacts with PIF4 to regulate high temperature-mediated hypocotyl elongation in response to blue light. Proc Natl Acad Sci U S A.

[14] Liu Y, Li X, Li K, Liu H, Lin C (2013). Multiple bHLH proteins form heterodimers to mediate CRY2-dependent regulation of flowering-time in Arabidopsis. PLoS Genet.

[15] Barrero JM, Downie AB, Xu Q, Gubler F (2014). A role for barley CRYPTOCHROME1 in light regulation of grain dormancy and germination. Plant Cell.

[16] Mao Z, Wei X, Li L, Xu P, Zhang J, Wang W, Guo T, Kou S, Wang W, Miao L (2021). Arabidopsis Cryptochrome 1 Controls Photomorphogenesis through Regulation of H2A.Z Deposition. Plant Cell.

[17] Liu H, Yu X, Li K, Klejnot J, Yang H, Lisiero D, Lin C (2008). Photoexcited CRY2 interacts with CIB1 to regulate transcription and floral initiation in Arabidopsis. Science.

[18] Zuo Z, Liu H, Liu B, Liu X, Lin C (2011). Blue light-dependent interaction of CRY2 with SPA1 regulates COP1 activity and floral initiation in Arabidopsis. Curr Biol.

[19] Meng Y, Li H, Wang Q, Liu B, Lin C (2013). Blue light-dependent interaction between cryptochrome2 and CIB1 regulates transcription and leaf senescence in soybean. Plant Cell.

[20] Kang CY, Lian HL, Wang FF, Huang JR, Yang HQ (2009). Cryptochromes, phytochromes, and COP1 regulate light-controlled stomatal development in Arabidopsis. Plant Cell.

[21] Mao J, Zhang YC, Sang Y, Li QH, Yang HQ (2005). From The Cover: A role for Arabidopsis cryptochromes and COP1 in the regulation of stomatal opening. Proc Natl Acad Sci U S A.

[22] D'Amico-Damiao V, Carvalho RF (1897). Cryptochrome-related abiotic stress responses in plants. Front Plant Sci.

[23] Wu L, Yang HQ (2010). CRYPTOCHROME 1 is implicated in promoting R protein-mediated plant resistance to Pseudomonas syringae in Arabidopsis. Mol Plant.

[24] D'Amico-Damiao V, Lucio JCB, Oliveira R, Gaion LA, Barreto RF, Carvalho RF (2021). Cryptochrome 1a depends on blue light fluence rate to mediate osmotic stress responses in tomato. J Plant Physiol.

[25] D'Amico-Damiao V, Dodd IC, Oliveira R, Lucio JCB, Rossatto DR, Carvalho RF (2021). Cryptochrome 1a of tomato mediates long-distance signaling of soil water deficit. Plant Sci.

[26] Hwang OJ, Back K (2021). Suppression of Rice Cryptochrome 1b Decreases Both Melatonin and Expression of Brassinosteroid Biosynthetic Genes Resulting in Salt Tolerance. Molecules.

[27] Xu P, Xiang Y, Zhu H, Xu H, Zhang Z, Zhang C, Zhang L, Ma Z (2009). Wheat cryptochromes: subcellular localization and involvement in photomorphogenesis and osmotic stress responses. Plant Physiol.

[28] Zhou T, Meng L, Ma Y, Liu Q, Zhang Y, Yang Z, Yang D, Bian M (2018). Overexpression of sweet sorghum cryptochrome 1a confers hypersensitivity to blue light, abscisic acid and salinity in Arabidopsis. Plant Cell Rep.

[29] Chen Z-W, Cao J-F, Zhang X-F, Shangguan X-X, Mao Y-B, Wang L-J, Chen X-Y (2017). Cotton genome: challenge into the polyploidy. Sci Bull.

[30] Wendel FJ, Grover C (2015). Taxonomy and evolution of the cotton genus, *Gossypium*.

[31] Wang K, Wendel JF, Hua J (2018). Designations for individual genomes and chromosomes in Gossypium. J Cotton Res.

[32] Paterson AH, Wendel JF, Gundlach H, Guo H, Jenkins J, Jin D, Llewellyn D, Showmaker KC, Shu S, Udall J (2012). Repeated polyploidization of Gossypium genomes and the evolution of spinnable cotton fibres. Nature.

[33] Wang K, Wang Z, Li F, Ye W, Wang J, Song G, Yue Z, Cong L, Shang H, Zhu S (2012). The draft genome of a diploid cotton *Gossypium raimondii*. Nat Genet.

[34] Udall JA, Long E, Hanson C, Yuan D, Ramaraj T, Conover JL, Gong L, Arick MA, Grover CE, Peterson DG (2019). De novo genome sequence assemblies of *Gossypium raimondii* and *Gossypium turneri*. G3 (Bethesda).

[35] Grover CE, Arick MA, Thrash A, Conover JL, Sanders WS, Peterson DG, Frelichowski JE, Scheffler JA, Scheffler BE, Wendel JF (2018). Insights into the evolution of the New World diploid cottons (*Gossypium*, Subgenus Houzingenia) based on genome sequencing. Genome Biol Evol.

[36] Li F, Fan G, Wang K, Sun F, Yuan Y, Song G, Li Q, Ma Z, Lu C, Zou C (2014). Genome sequence of the cultivated cotton *Gossypium arboreum*. Nat Genet.

[37] Du X, Huang G, He S, Yang Z, Sun G, Ma X, Li N, Zhang X, Sun J, Liu M (2018). Resequencing of 243 diploid cotton accessions based on an updated A genome identifies the genetic basis of key agronomic traits. Nat Genet.

[38] Huang G, Wu Z, Percy RG, Bai M, Li Y, Frelichowski JE, Hu J, Wang K, Yu JZ, Zhu Y (2020). Genome sequence of Gossypium herbaceum and genome updates of Gossypium arboreum and Gossypium hirsutum provide insights into cotton A-genome evolution. Nat Genet.

[39] Grover CE, Pan M, Yuan D, Arick MA, Hu G, Brase L, Stelly DM, Lu Z, Schmitz RJ, Peterson DG (2020). The *Gossypium longicalyx* genome as a resource for cotton breeding and evolution. G3.

[40] Cai Y, Cai X, Wang Q, Wang P, Zhang Y, Cai C, Xu Y, Wang K, Zhou Z, Wang C (2020). Genome sequencing of the Australian wild diploid species Gossypium australe highlights disease resistance and delayed gland morphogenesis. Plant Biotechnol J.

[41] Zhang T, Hu Y, Jiang W, Fang L, Guan X, Chen J, Zhang J, Saski CA, Scheffler BE, Stelly DM (2015). Sequencing of allotetraploid cotton (Gossypium hirsutum L. acc. TM-1) provides a resource for fiber improvement. Nat Biotechnol.

[42] Chen ZJ, Sreedasyam A, Ando A, Song Q, De Santiago LM, Hulse-Kemp AM, Ding M, Ye W, Kirkbride RC, Jenkins J (2020). Genomic diversifications of five Gossypium allopolyploid species and their impact on cotton improvement. Nat Genet.

[43] Li F, Fan G, Lu C, Xiao G, Zou C, Kohel RJ, Ma Z, Shang H, Ma X, Wu J (2015). Genome sequence of cultivated Upland cotton (Gossypium hirsutum TM-1) provides insights into genome evolution. Nat Biotechnol.

[44] Liu X, Zhao B, Zheng HJ, Hu Y, Lu G, Yang CQ, Chen JD, Chen JJ, Chen DY, Zhang L (2015). Gossypium barbadense genome sequence provides insight into the evolution of extra-long staple fiber and specialized metabolites. Sci Rep.

[45] Yuan D, Tang Z, Wang M, Gao W, Tu L, Jin X, Chen L, He Y, Zhang L, Zhu L (2015). The genome sequence of Sea-Island cotton (Gossypium barbadense) provides insights into the allopolyploidization and development of superior spinnable fibres. Sci Rep.

[46] Wang M, Tu L, Yuan D, Zhu D, Shen C, Li J, Liu F, Pei L, Wang P, Zhao G (2019). Reference genome sequences of two cultivated allotetraploid cottons, Gossypium hirsutum and Gossypium barbadense. Nat Genet.

[47] Hu Y, Chen J, Fang L, Zhang Z, Ma W, Niu Y, Ju L, Deng J, Zhao T, Lian J (2019). Gossypium barbadense and Gossypium hirsutum genomes provide insights into the origin and evolution of allotetraploid cotton. Nat Genet.

[48] Yang Z, Ge X, Yang Z, Qin W, Sun G, Wang Z, Li Z, Liu J, Wu J, Wang Y (2019). Extensive intraspecific gene order and gene structural variations in upland cotton cultivars. Nat Commun.

[49] Udall JA, Long E, Ramaraj T, Conover JL, Yuan D, Grover CE, Gong L, Arick MA, Masonbrink RE, Peterson DG (2019). The genome sequence of* Gossypioides kirkii* illustrates a descending dysploidy in plants. Front Plant Sci.

[50] Blazquez M, Koornneef M, Putterill J (2001). Flowering on time: genes that regulate the floral transition. Workshop on the molecular basis of flowering time control. EMBO Rep.

[51] Kleine T, Lockhart P, Batschauer A (2003). An Arabidopsis protein closely related to Synechocystis cryptochrome is targeted to organelles. Plant J.

[52] Yu X, Shalitin D, Liu X, Maymon M, Klejnot J, Yang H, Lopez J, Zhao X, Bendehakkalu KT, Lin C (2007). Derepression of the NC80 motif is critical for the photoactivation of Arabidopsis CRY2. Proc Natl Acad Sci U S A.

[53] Hernandez-Garcia CM, Finer JJ (2014). Identification and validation of promoters and cis-acting regulatory elements. Plant Sci.

[54] Lescot M, Dehais P, Thijs G, Marchal K, Moreau Y, Van de Peer Y, Rouze P, Rombauts S (2002). PlantCARE, a database of plant cis-acting regulatory elements and a portal to tools for in silico analysis of promoter sequences. Nucleic Acids Res.

[55] Kleiner O, Kircher S, Harter K, Batschauer A (1999). Nuclear localization of the Arabidopsis blue light receptor cryptochrome 2. Plant J.

[56] Yu X, Liu H, Klejnot J, Lin C (2010). The cryptochrome blue light receptors. Arabidopsis Book.

[57] Mishra S, Khurana JP (2017). Emerging roles and new paradigms in signaling mechanisms of plant cryptochromes. Crit Rev Plant Sci.

[58] Kami C, Lorrain S, Hornitschek P, Fankhauser C (2010). Light-regulated plant growth and development. Curr Top Dev Biol.

[59] Yang Z, Liu B, Su J, Liao J, Lin C, Oka Y (2017). Cryptochromes Orchestrate Transcription Regulation of Diverse Blue Light Responses in Plants. Photochem Photobiol.

[60] Sharma P, Chatterjee M, Burman N, Khurana JP (2014). Cryptochrome 1 regulates growth and development in Brassica through alteration in the expression of genes involved in light, phytohormone and stress signalling. Plant Cell Environ.

[61] Lopez L, Carbone F, Bianco L, Giuliano G, Facella P, Perrotta G (2012). Tomato plants overexpressing cryptochrome 2 reveal altered expression of energy and stress-related gene products in response to diurnal cues. Plant Cell Environ.

[62] Pooam M, Dixon N, Hilvert M, Misko P, Waters K, Jourdan N, Drahy S, Mills S, Engle D, Link J (2021). Effect of temperature on the Arabidopsis cryptochrome photocycle. Physiol Plant.

[63] Yu J, Jung S, Cheng CH, Ficklin SP, Lee T, Zheng P, Jones D, Percy RG, Main D (2014). CottonGen: a genomics, genetics and breeding database for cotton research. Nucleic Acids Res.

[64] Wong DC, Schlechter R, Vannozzi A, Holl J, Hmmam I, Bogs J, Tornielli GB, Castellarin SD, Matus JT (2016). A systems-oriented analysis of the grapevine R2R3-MYB transcription factor family uncovers new insights into the regulation of stilbene accumulation. DNA Res.

[65] Mistry J, Finn RD, Eddy SR, Bateman A, Punta M (2013). Challenges in homology search: HMMER3 and convergent evolution of coiled-coil regions. Nucleic Acids Res.

[66] Poole RL (2007). The TAIR database. Methods Mol Biol.

[67] Letunic I, Bork P (2018). 20 years of the SMART protein domain annotation resource. Nucleic Acids Res.

[68] Finn RD, Coggill P, Eberhardt RY, Eddy SR, Mistry J, Mitchell AL, Potter SC, Punta M, Qureshi M, Sangrador-Vegas A (2016). The Pfam protein families database: towards a more sustainable future. Nucleic Acids Res.

[69] Chen C, Chen H, Zhang Y, Thomas HR, Frank MH, He Y, Xia R (2020). TBtools: An Integrative Toolkit Developed for Interactive Analyses of Big Biological Data. Mol Plant.

[70] Hu B, Jin J, Guo AY, Zhang H, Luo J, Gao G (2015). GSDS 2.0: an upgraded gene feature visualization server. Bioinformatics.

[71] Wilkins MR, Gasteiger E, Bairoch A, Sanchez JC, Williams KL, Appel RD, Hochstrasser DF (1999). Protein identification and analysis tools in the ExPASy server. Methods Mol Biol.

[72] Katoh K, Standley DM (2013). MAFFT multiple sequence alignment software version 7: improvements in performance and usability. Mol Biol Evol.

[73] Rozas J, Ferrer-Mata A, Sanchez-DelBarrio JC, Guirao-Rico S, Librado P, Ramos-Onsins SE, Sanchez-Gracia A (2017). DnaSP 6: DNA Sequence Polymorphism Analysis of Large Data Sets. Mol Biol Evol.

[74] Tamura K, Stecher G, Peterson D, Filipski A, Kumar S (2013). MEGA6: Molecular Evolutionary Genetics Analysis version 6.0. Mol Biol Evol.

[75] Zhao B, Cao JF, Hu GJ, Chen ZW, Wang LY, Shangguan XX, Wang LJ, Mao YB, Zhang TZ, Wendel JF (2018). Core cis-element variation confers subgenome-biased expression of a transcription factor that functions in cotton fiber elongation. New Phytol.

[76] Kim D, Langmead B, Salzberg SL (2015). HISAT: a fast spliced aligner with low memory requirements. Nat Methods.

[77] Pertea M, Kim D, Pertea GM, Leek JT, Salzberg SL (2016). Transcript-level expression analysis of RNA-seq experiments with HISAT StringTie and Ballgown. Nat Protoc.

[78] Risso D, Ngai J, Speed TP, Dudoit S (2014). Normalization of RNA-seq data using factor analysis of control genes or samples. Nat Biotechnol.

[79] Wang M, Wang Q, Zhang B (2013). Evaluation and selection of reliable reference genes for gene expression under abiotic stress in cotton (Gossypium hirsutum L.). Gene.

[80] Zhang Y, Parmigiani G, Johnson WE. ComBat-seq: batch effect adjustment for RNA-seq count data. NAR Genom Bioinform. 2020;2(3):lqaa078. 10.1093/nargab/lqaa078 PMC7518324 33015620

[81] Shangguan X, Yang Q, Wu X, Cao J (2021). Function analysis of a cotton R2R3 MYB transcription factor GhMYB3 in regulating plant trichome development. Plant Biol (Stuttg).

[82] You Q, Xu WY, Zhang K, Zhang LW, Yi X, Yao DX, Wang CC, Zhang XY, Zhao XH, Provart NJ (2017). ccNET: Database of co-expression networks with functional modules for diploid and polyploid *Gossypium*. Nucleic Acids Res.

[83] Shan CM, Shangguan XX, Zhao B, Zhang XF, Chao LM, Yang CQ, Wang LJ, Zhu HY, Zeng YD, Guo WZ (2014). Control of cotton fibre elongation by a homeodomain transcription factor GhHOX3. Nat Commun.

[84] Cao J-F, Huang J-Q, Liu X, Huang C-C, Zheng Z-S, Zhang X-F, Shangguan X-X, Wang L-J, Zhang Y-G, Wendel JF (2020). Genome-wide characterization of the GRF family and their roles in response to salt stress in Gossypium. BMC Genomics.

[85] Cao J-F, Zhao B, Huang C-C, Chen Z-W, Zhao T, Liu H-R, Hu G-J, Shangguan X-X, Shan C-M, Wang L-J (2020). The miR319-Targeted GhTCP4 Promotes the Transition from Cell Elongation to Wall Thickening in Cotton Fiber. Mol Plant.

[86] Zhang X, Cao J, Huang C, Zheng Z, Liu X, Shangguan X, Wang L, Zhang Y, Chen Z (2021). Characterization of cotton ARF factors and the role of GhARF2b in fiber development. BMC Genomics.

[87] Livak KJ, Schmittgen TD (2001). Analysis of relative gene expression data using real-time quantitative PCR and the 2(T)(-Delta Delta C) method. Methods.

