# Supplementary material for: Comprehensive identification and expression analysis of CRY gene family in Gossypium
Source: BMC Genomics. 2022 Mar 24;23:231. doi: 10.1186/s12864-022-08440-9 (PMC8952943; doi:10.1186/s12864-022-08440-9)
Supplement: Supplementary file 1 — Additional file 1: Table S1. Ka, Ks and Ka/Ks analyses of GhCRY from the A and D subgenomes compared with their corresponding progenitor homoeologs. [file 12864_2022_8440_MOESM1_ESM.docx]

**Additional file 1: Table S1.** Ka, Ks and Ka/Ks analyses of *GhCRY* from the A and D subgenomes compared with their corresponding progenitor homoeologs.

| **Locus Name** | **Chrom** | **Locus Name** | **Chrom** | **Ka** | **Ks** | **Ka/Ks** |
| --- | --- | --- | --- | --- | --- | --- |
| Gh_A02G0384 | A02 | Cotton_A_22452 | CA_chr5 | 0.0030 | 0.0000 | 2.0000 |
| Gh_D02G0436 | D02 | Gorai.005G050000.1 | Chr5 | 0.0040 | 0.0191 | 0.2094 |
| Gh_A03G0120 | A03 | Cotton_A_17162 | CA_chr7 | 0.0035 | 0.0185 | 0.1892 |
| Gh_D03G1520 | D03 | Gorai.003G166800.1 | Chr3 | 0.0052 | 0.0154 | 0.3377 |
| Gh_A05G1941 | A05 | Cotton_A_05062 | CA_chr3 | 0.0006 | 0.0084 | 0.0714 |
| Gh_D05G2172 | D05 | Gorai.009G236700.1 | Chr9 | 0.0026 | 0.0106 | 0.2453 |
| Gh_A05G2282 | A05 | Cotton_A_23627 | CA_chr1 | 0.0013 | 0.0043 | 0.3023 |
| Gh_D05G2543 | D05 | Gorai.009G280900.1 | Chr9 | 0.0019 | 0.0172 | 0.1105 |
| Gh_A06G0969 | A06 | Cotton_A_31677 | CA_chr8 | 0.0024 | 0.0081 | 0.2963 |
| Gh_D06G1145 | D06 | Gorai.010G124000.1 | Chr10 | 0.0260 | 0.0386 | 0.6736 |
| Gh_A06G1059 | A06 | Cotton_A_40982 | CA_chr8 | 0.0182 | 0.0231 | 0.7879 |
| Gh_D06G2339 | D06 | Gorai.010G143600.1 | Chr10 | 0.0024 | 0.0027 | 0.8889 |
| Gh_A09G2012 | A09 | Cotton_A_10622 | CA_chr11 | 0.0026 | 0.0045 | 0.5778 |
| Gh_D09G2225 | D09 | Gorai.006G253700.1 | Chr6 | 0.0053 | 0.0094 | 0.5638 |
| Gh_A11G1040 | A11 | Cotton_A_23295 | CA_chr4 | 0.0028 | 0.0062 | 0.4516 |
| Gh_D11G1195 | D11 | Gorai.007G127800.1 | Chr7 | 0.0038 | 0.0154 | 0.2468 |
| Gh_A12G2401 | A12 | Cotton_A_01388 | CA_chr6 | 0.0045 | 0.0081 | 0.5556 |
| Gh_D12G2528 | D12 | Gorai.008G284900.1 | Chr8 | 0.0019 | 0.0163 | 0.1166 |
