# Supplementary figures and images for: Comprehensive identification and expression analysis of CRY gene family in Gossypium
Source: BMC Genomics. 2022 Mar 24;23:231. doi: 10.1186/s12864-022-08440-9 (PMC8952943; doi:10.1186/s12864-022-08440-9)

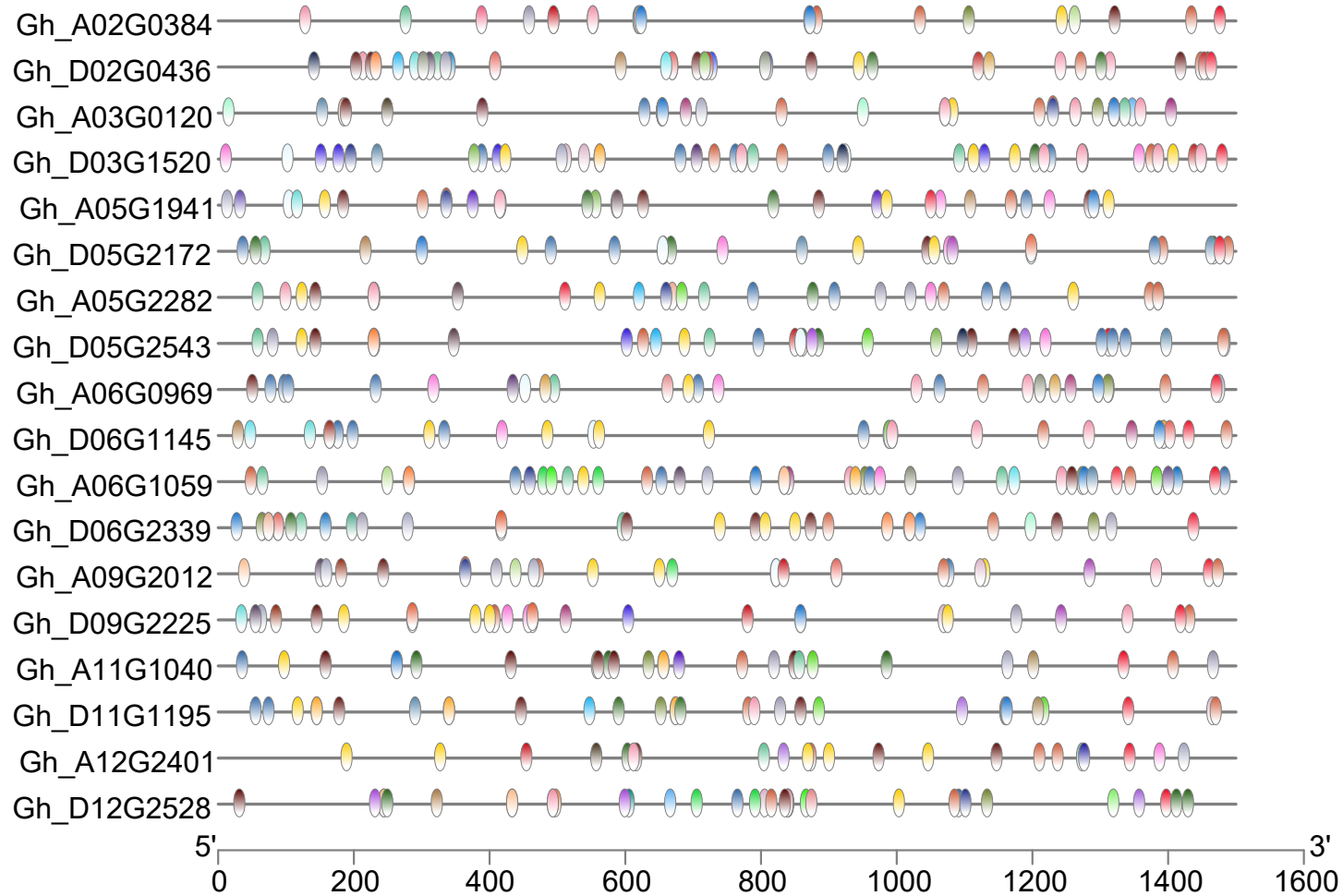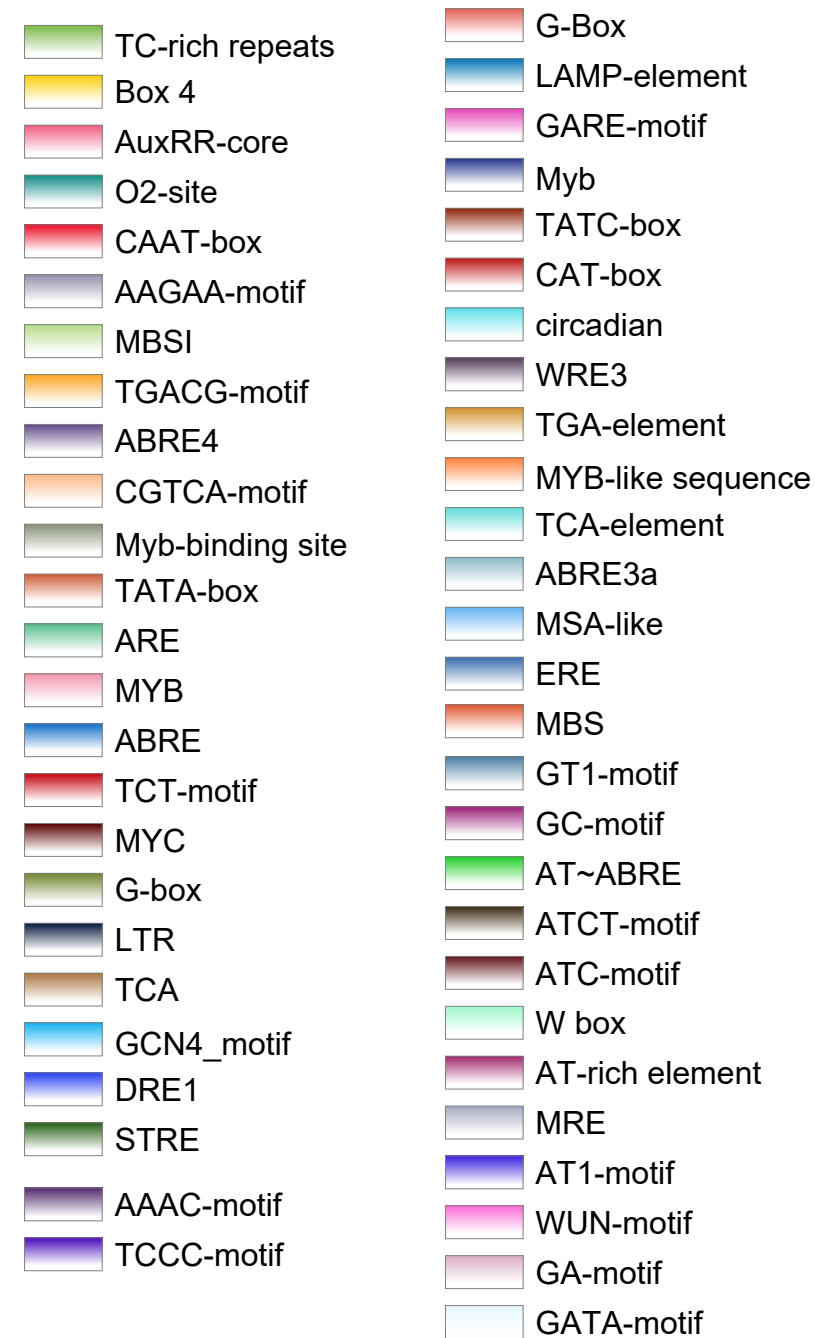

Supplement: Supplementary file 2 — Additional file 2: Figure S1. Cis-elements in promoter regions of GhCRY genes. [file 12864_2022_8440_MOESM2_ESM.pdf]

**A***Gh\_A05G1941*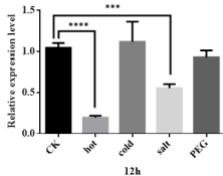**B***Gh\_A05G2282*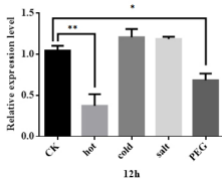**C***Gh\_A06G1059*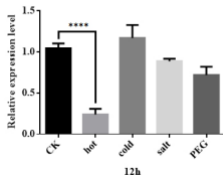**D***Gh\_A12G2401*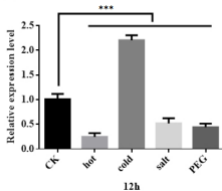

Supplement: Supplementary file 3 — Additional file 3: Figure S2. Expression profile analyses of four GhCRY genes under different stress treatments at the time points of 12h analyzed by qRT-PCR.(A): Gh_A05G1941;(B):Gh_A05G2282; (C):Gh_A06G1059; (D): Gh_A12G2401. [file 12864_2022_8440_MOESM3_ESM.pdf]
