# Supplementary material for: Comprehensive identification and expression analysis of CRY gene family in Gossypium
Source: BMC Genomics. 2022 Mar 24;23:231. doi: 10.1186/s12864-022-08440-9 (PMC8952943; doi:10.1186/s12864-022-08440-9)
Supplement: Supplementary file 4 — Additional file 4: Table S2. List of forward and reverse primers used for qRT-PCR analyses. [file 12864_2022_8440_MOESM4_ESM.docx]

**TABLE S2 |** List of forward and reverse primers used for qRT-PCR analyses.

| Gene Name | Forward Primer (5'to3') | Reverse Primer (5'to3') |
| --- | --- | --- |
| *Gh_A06G1059* | ATCCCAACATGCCATCCG | ACCGGATCCTGTTTATACCCG |
| *Gh_A05G1941* | GCTACAACTCGGCGATACGA | CAACTCCGCCATCCCTTTCT |
| *Gh_A05G2282* | AGGCCTCTTCTCGGACATCT | GCCACCGACGGACATATTCT |
| *Gh_A12G2401* | TTCGCCTCTTCTTCCTGCAAC | TCGTGCTGTTCCTTCCAAGT |
| *GhHIS3* | GAAGCCTCATCGATACCGTC | CTACCACTACCATCATGG |
